# Chinese-like Strain of Porcine Epidemic Diarrhea Virus, Thailand

**DOI:** 10.3201/eid1507.081256

**Published:** 2009-07

**Authors:** Suphasawatt Puranaveja, Pariwat Poolperm, Preeda Lertwatcharasarakul, Sawang Kesdaengsakonwut, Alongkot Boonsoongnern, Kitcha Urairong, Pravina Kitikoon, Porjit Choojai, Roongtham Kedkovid, Komkrich Teankum, Roongroje Thanawongnuwech

**Affiliations:** Chulalongkorn University, Bangkok, Thailand (S. Puranaveja, S. Kesdaengsakonwut, P. Kitikoon, P. Choojai, R. Kedkovid, K. Teankum, R. Thanawongnuwech); Kasetsart University**,** Kamphangsan**,** Thailand (P. Poolperm, P. Lertwatcharasarakul, A. Boonsoongnern, K. Urairong)

**Keywords:** Porcine epidemic diarrhea, virus, molecular epidemiology, pathology, Thailand, dispatch

## Abstract

Since late 2007, several outbreaks of porcine epidemic diarrhea virus (PEDV) infection have emerged in Thailand. Phylogenetic analysis places all Thai PEDV isolates during the outbreaks in the same clade as the Chinese strain JS-2004-2. This new genotype PEDV is prevailing and currently causing sporadic outbreaks in Thailand.

Porcine epidemic diarrhea virus (PEDV), first recognized in 1977 (*1*), is an enveloped, single-stranded RNA virus belonging to the family *Coronaviridae*. The PEDV genome contains genes for the following proteins: pol1 (P1), spike (S) (180–220 kDa), envelope (E), membrane (M) (27–32 kDa), and nucleocapsid (N) (55–58 kDa) (*2*). The M protein is a structural membrane glycoprotein, which plays an important role in the assembly process; the S surface glycoprotein harbors the specific host cell receptor binding sites (*3*).

During late 2007, the PED outbreak appeared first in Nakornpathom province before spreading throughout the country. Pig losses from the recent PED outbreaks were extensive. Obvious clinical signs were severe diarrhea (Figure 1, panel A) and dehydration with milk curd vomitus in suckling piglets. Most of the affected farms reported the disease first in farrowing barns and subsequently lost 100% of newborn piglets. Pigs of all ages were affected and exhibited degrees of diarrhea and inappetite, which varied by their ages. Boars and sows had mild diarrhea and anorexia for a few days and recovered within a week. In piglets that died, the small intestinal wall was congested and intestinal contents were watery with undigested milk curd (Figure 1, panel B). Segmental enteritis was indicated by segmental disappearance of intestinal lacteal caused by malabsorption in affected intestinal parts (Figure 1, panels C and D). Atrophic enteritis, characterized by blunting of the intestinal villi and sloughing of intestinal epithelium, occurred in all affected piglets (Figure 2, panel A). Immunohistochemical tests, performed by using monoclonal anti-PEDV S protein (JBT Biotechnology Laboratory, Seoul, South Korea), demonstrated dark brown staining in intestinal epithelial cells (Figure 2, panel B). Massive feedback of piglet feces and minced piglet guts to gestating sows was recommended by local veterinary practitioners to prime the sow’s immune response and pass protective immunity to the piglets. At affected farms, the outbreak lasted <3 weeks.

**Figure 1:**
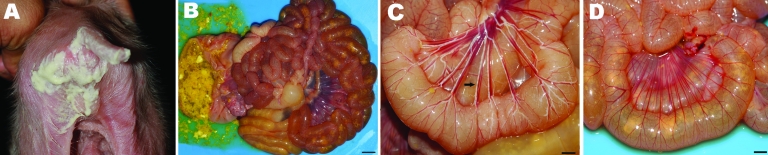
A) A suckling piglet with severe diarrhea and dehydration. B) Severe catarrhal enteritis with congestion (scale bar = 1 cm). C) Intestinal lacteals (arrow) grossly demonstrating normal absorption capacity of the intestinal villi in a normal piglet (scale bar = 0.5 cm). D) Disappearance of intestinal lacteals demonstrating malabsorption syndrome of the intestinal villi in the infected piglet (scale bar = 0.5 cm).

**Figure 2:**
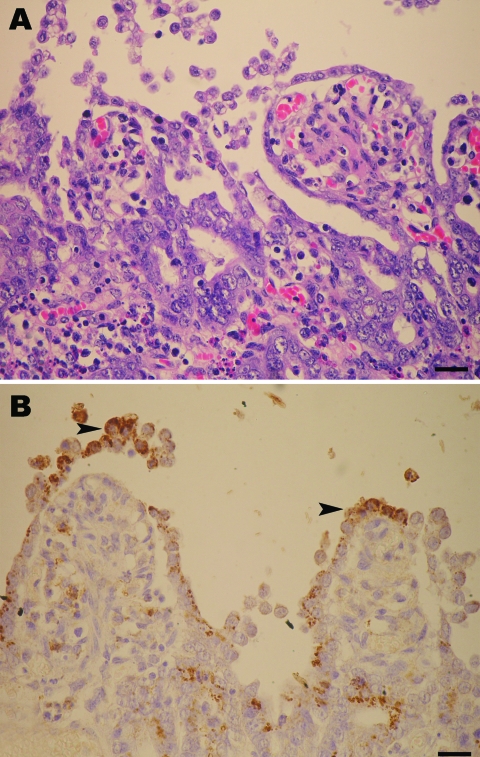
A) Marked shortening and blunting of the intestinal villi (scale bar = 25 µm). B) Intestinal epithelial cells expressing porcine epidemic diarrhea virus antigen (arrowheads) in the cytoplasm (colon), visible as brown staining (scale bar = 25 µm).

## The Study

Samples from 8 provinces (24 farms) in Thailand from December 2007 through March 2008 were submitted to the veterinary diagnostic laboratories of Kasetsart University and Chulalongkorn University. A total of 33 porcine samples were confirmed as positive for PEDV by reverse transcription–PCR (RT-PCR) (*4*) before virus isolation (Table). Published primers (*5*) were used for generating the PEDV 651-bp partial S gene. Primers were designed to amplify the PEDV M gene and yielded the amplified product of 715 bp on the basis of CV777 and Br1/87. Products were purified by using a QIAquick Gel Extraction Kit (QIAGEN, Hilden, Germany) and were sequenced by 1st BASE Pte Ltd (Singapore).

**Table:** Thirty-three PEDV isolates obtained from 8 provinces in Thailand during outbreaks, 2007–2008*

Isolate no.	Isolate name	Date isolated	Geographic origin	GenBank accession nos., M/S gene
1	07NP01	2007 Dec	Nakornpathom (W)	FJ196165/FJ196196
2	08CB01	2008 Jan	Chonburi (E)	FJ196166/FJ196197
3	08RB01	2008 Jan	Ratchaburi (W)	FJ196182/FJ196213
4	08RB02	2008 Jan	Ratchaburi (W)	FJ196183/FJ196214
5	08CB02	2008 Jan	Chonburi (E)	FJ196167/FJ196198
6	08RB03	2008 Jan	Ratchaburi (W)	FJ196184/FJ196215
7	08NP02	2008 Jan	Nakornpathom (W)	FJ196173/FJ196204
8	08NP03	2008 Jan	Nakornpathom (W)	FJ196174/FJ196205
9	08PB01	2008 Jan	Pretchaburi (S)	FJ196180/FJ196211
10	08NP04	2008 Jan	Nakornpathom (W)	FJ196175/FJ196206
11	08CB03	2008 Jan	Chonburi (E)	FJ196168/FJ196199
12	08NP05	2008 Feb	Nakornpathom (W)	FJ196176/FJ196207
13	08RB04	2008 Feb	Ratchaburi (W)	FJ196185/FJ196216
14	08CB04	2008 Feb	Chonburi (E)	FJ196169/FJ196200
15	08RB05	2008 Feb	Ratchaburi (W)	FJ196186/FJ196216
16	08RB06	2008 Feb	Ratchaburi (W)	FJ196187/FJ196217
17	08CB05	2008 Mar	Chonburi (E)	FJ196170/FJ196201
18	08NP06	2008 Mar	Nakornpathom (W)	FJ196177/FJ196208
19	08NP07	2008 Mar	Nakornpathom (W)	FJ196178/FJ196209
20	08UB01	2008 Mar	Ubon Ratchathani (NE)	FJ196189/FJ196220
21	08CC01	2008 Mar	Chachoengsao (E)	FJ196172/FJ196203
22	08CB06	2008 Mar	Chonburi (E)	FJ196171/FJ196202
23	08RB07	2008 Mar	Ratchaburi (W)	FJ196188/FJ196219
24	08NP08	2008 Mar	Nakornpathom (W)	FJ196179/FJ196210
25	08PC01	2008 Mar	Prachinburi (E)	FJ196181/FJ196212
26	KU01CB08	2008 Jan	Chonburi (E)	FJ196190/FJ196221
27	KU02NK08	2008 Feb	Nongkhai (NE)	–/FJ196222
28	KU03CB08	2008 Feb	Chonburi (E)	FJ196191/FJ196223
29	KU04RB08	2008 Feb	Ratchaburi (W)	FJ196192/FJ196224
30	KU05CB08	2008 Mar	Chonburi (E)	FJ196193/FJ196225
31	KU06RB08	2008 Mar	Ratchaburi (W)	FJ196194/FJ196226
32	KU07RB08	2008 Mar	Ratchaburi (W)	FJ196195/FJ196227
33	KU08RB08	2008 Mar	Ratchaburi (W)	–/FJ196228

*PEDV, porcine epidemic diarrhea virus; E, eastern, W, western, NE, northeastern; S, southern.

Nucleotide and deduced amino acid sequences of the 33 PEDV isolates were aligned, edited, and analyzed with ClustalX version 1.83, Bioedit version 7.0.5.2, and MegAlign software (DNAStar Inc., Madison, WI, USA), respectively. Phylogenetic trees were generated by using partial S and full-length M genes, including the deduced amino acid sequences with selected reference PEDV strains, by applying the Jotun Hein method in the MegAlign software. To assess the relative support for each clade, bootstrap values were calculated from 1,000 replicate analyses.

The M gene sequence analysis of 31 PEDV isolates obtained in Thailand indicated that the nucleotide sequence of the entire M gene was highly conserved. All recent PEDV isolates in Thailand had 99.3%–100% nucleotide homology. The lowest sequence identity (96.5%) was with the Chinese strain, EF185992/LZC, and the highest sequence identity (99.2%–99.7%) was with the Chinese strain, JS-2004-2, and concurrent isolates from the National Institute of Animal Health, Thailand, M_NIAH 07-08 (data not shown).

All 33 PEDV isolates had 97.0%–98.8% DNA sequence identities of the S gene with each other. Our findings demonstrated that the recent PEDV isolates in Thailand were genetically diverse in their S genes either within their group or with the reference strains. These point mutations may lead to genetic diversity among these isolates. The lowest sequence identity (95.7%) was with the Korean strain, Chinju99. Similar to the M gene results, the highest sequence identity of the S gene (98.6%) was with the Chinese strain, JS-2004-2 (data not shown).

Three major clusters based on the phylogenetic relation of the nucleotide sequences of the M gene (Appendix Figure panel A) were detected. The first cluster comprised all Thai isolates in 2007–2008 and 3 Chinese strains (JS-2004-2, LJB/03, and QH). The second cluster consisted of 2 Korean strains (KPEDV-9 and Chinju99), 1 Japanese strain (Jme2), 2 Chinese isolates (HN-XYYYP-2007 and YM 2007), and a previous Thai isolate in 2004 (M_NIAH_04). The third cluster contained Br1/87 (CV777), 2 Russian isolates, and 1 Chinese strain (LZC).

On the basis of the phylogenetic relation of the nucleotide sequences of the partial S gene, 3 groups were identified (Appendix Figure panel B). Group 1 comprised all recent Thai PEDV and the Chinese PEDV strains isolated in 2003–2004 (JS-2004-2 and LJB/03). Notably, a Thai isolate, 08NP04, was very similar to JS-2004-2. Group 2 comprised Br1/87 (LZC) and CV777. Group 3 consisted of the Korean isolates (Spk1 and Chinju99).

The recent Thai PEDV strain was closely related to the isolates from China, JS-2004-2 and LJB/03. There was no insertion or deletion in the M gene of the recent Thai isolates except for minor point mutations. M_NIAH/04, isolated in 2004 in Thailand, had a slightly different nucleotide sequence from the recent Thai isolates (R. Thanawongnuwech, unpub. data).

Our results indicated that the recent Thai PEDV isolates clustered in the same group were highly homologous with the Chinese strains, JS-2004-2 and LJB/03. They were responsible for the recent PED outbreak in Thailand and able to produce pathologic effects similar to the Chinese isolates (*6*). Notably, 08NP04, isolated 4 months after the first outbreak, had the highest identity to JS-2004-2. Also, 08NP04 and 07NP01 (the first isolates in 2007) originated in the same geographic area. The Chinese-like strain of the virus might have gained entry into Thailand via unknown routes as early as December 2007. In addition, rendering trucks traveling from farm to farm might have encouraged widespread transmission of the disease. Structural differences in the partial S gene could help elucidate pathogenesis and antigenic structures of the recent PEDV isolates because S glycoproteins are responsible for inducing the virus neutralizing antibodies and known to be highly conserved in PEDV strains (*7*). Continuing investigation of PEDV isolates will contribute to the prevention and control of this virus.

## Conclusions

The phylogenetic relationship of the Thai PEDV strain indicated that the recent Thai PEDV isolates differed genetically from previous Thai isolates. Despite precautions, sporadic outbreaks continue to occur. In addition, disease transmission frequently occurs due to the purchase of new stock with improper gilt acclimatization and biosecurity. Immunity induced through vaccination, currently unavailable in Thailand, does not provide lifelong protection from this virus. However, vaccination is recommended to encourage specific immunity to PEDV in all stock when an acute outbreak occurs. Undoubtedly, effective biosecurity is a key management tool for PED prevention and control. Our data suggested that all recent Thai PEDV isolates are genetically similar to the Chinese isolates identified in 2004. Further analysis of the entire S gene of PEDV and of other isolates in neighboring countries is needed to show the molecular epidemiology of the Chinese-like strain.

## Supplementary Material

Appendix Figure Phylogenetic trees generated on the basis of nucleotide of the M gene region (A) and the partial S gene region (B). Trees constructed with neighbor-joining method by using MEGA 3.1 (DNAStar Inc., Madison, WI, USA). Horizontal branch lengths are proportional to genetic distances between Porcine epidemic diarrhea virus (PEDV) strains.
