# Supplementary figures and images for: Chinese-like Strain of Porcine Epidemic Diarrhea Virus, Thailand
Source: Emerg Infect Dis. 2009 Jul;15(7):1112–5. doi: 10.3201/eid1507.081256 (PMC2744260; doi:10.3201/eid1507.081256)

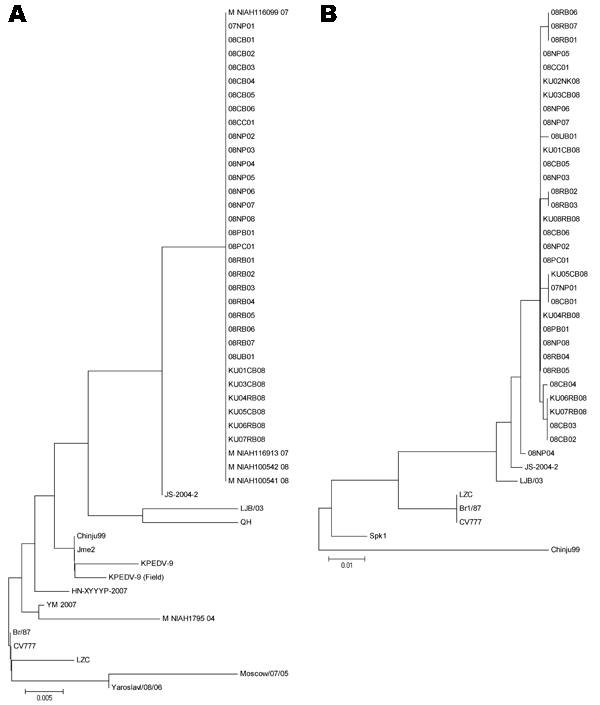

Supplement: Appendix Figure — Phylogenetic trees generated on the basis of nucleotide of the M gene region (A) and the partial S gene region (B). Trees constructed with neighbor-joining method by using MEGA 3.1 (DNAStar Inc., Madison, WI, USA). Horizontal branch lengths are proportional to genetic distances between Porcine epidemic diarrhea virus (PEDV) strains. [file 08-1256_appF-s1.gif]
